# China's growing contribution to sepsis research from 1984 to 2014: A bibliometric study

**DOI:** 10.1097/MD.0000000000007275

**Published:** 2017-06-23

**Authors:** Kai Zhang, Jialian Zhao, Lihua Chu, Yue Jin, Baoli Cheng, Guohao Xie, Yan Wang, Xiangming Fang

**Affiliations:** Department of Anesthesiology, the First Affiliated Hospital, School of Medicine, Zhejiang University, Hangzhou, China.

**Keywords:** bibliometric analysis, China, PubMed, sepsis, Web of Science

## Abstract

**Background:**

The aim of this study was to analyze the global trends of sepsis research and investigate China's contribution by comparing the quantity and quality of sepsis-related publications.

**Methods:**

Sepsis-related publications were main retrieved from the Web of Science online database by using the keywords “sepsis” or “septic shock.” Bibliometric indicators, including the number of publications, total citation frequency, citation frequency per paper, h-index, research type, orientation, organization, author, journal, and funding support were analyzed.

**Results:**

From 1984 to 2014, a total of 70,564 papers regarding sepsis research were published worldwide. 3.60% of which were contributed by authors from Chinese institutions. Although this contribution was less than the Unites States, Germany, England, France or Japan, a notable trend of increase in a number of scientific publications (*r*^2^  =  0.57, *P* < .001), with an average annual growth rate of 20.78%, was observed. The quality of the publications was relatively low in China. Basic research was a clear dominant representation, accounting for 50.46% of China's sepsis research. The research centers in China were mainly located in Beijing (5 centers), Shanghai (4 centers), and Hong Kong (3 centers). The National Natural Science Foundation of China supported the most Chinese sepsis researches.

**Conclusion:**

Global sepsis research developed swiftly during the 1984 to 2014 period. The USA was in the forefront of sepsis research. Although the data indicated that China had a large increasing rate of publications, there was a considerable gap in the quality of articles between China and other developed countries.

## Abstract 2

Supplemental Digital Content is available in the text

## 1 Introduction

Sepsis, a syndrome with physiologic, pathologic, and biochemical abnormalities induced by infection, is a major public health concern with high morbidity, mortality, and cost.^[1–3]^ It was first proposed and defined in the first Consensus Conference for Sepsis Definitions held by American College of Chest Physicians/Society of Critical Care Medicine (ACCP/SCCM) in 1991, which marked a new era for mechanisms, diagnoses and treatments in sepsis research.^[4]^ Up to now, countless specialists and researchers have devoted themselves to this field in order to get a better understanding in mechanism of sepsis and develop new methods for the diagnosis and treatment, and their research results all come to publications in the end. China, with the growth of the overall economy and scientific research strength, also make great achievements in sepsis research. But the global and China's development trend regarding sepsis has not been well studied yet.

Bibliometrics, a well-established research method in information science, is often used for statistical analysis in written publications.^[5]^ This scientific and quantitative method of analysis cannot only provide quantitative information about research environment, but also evaluate the quality of research by citation analysis, through which we can characterize research progress, explore the impact of a discipline, predict the development of a field, and give guidance to clinical and basic research.^[6,7]^ Much bibliometric research has been carried out and has made remarkable contributions to the development of modern medicine in the areas of primary care,^[8]^ neurology,^[9]^ respiratory medicine,^[10]^ urology,^[11]^ pathology,^[12]^ and cancer.^[13]^

This bibliometric study was aimed to analyze the global sepsis research trends and evaluate China's growing contribution to sepsis research by comparing the quantity and quality of sepsis-related publications during the 1984 to 2014 periods.

## 2 Methods

### 2.1 Sources of the data

Sepsis-related articles published between 1984 and 2014 were retrieved from the Web of Science (WOS) online database, including the Science Citation Index Expanded (SCIE), Conference Proceeding Citation Index-Science (CPCIS), Current Chemical Reactions (CCR)-Expanded and Index Chemical (IC). The journal impact factors (IF) complied with the standard of ISI Web of Knowledge Journal Citation Reports 2015 database. Foundation data from China were derived from the National Natural Science Foundation of China (NSFC) website. Additionally, research types, including basic research, randomized controlled trials (RCTs), clinical trials, and case reports, were retrieved from the PubMed database. All data were obtained on February 22, 2016. All the data were downloaded from the public databases and there were no ethical questions about it, therefore the ethical approval was unnecessary.

### 2.2 Search strategy

#### 2.2.1 Data from WOS

The search terms were: Theme  =  ((sepsis) or (septic shock)) AND publishing year  =  (1984–2014). Refining: literature type  =  (Article or Letter or Review).

#### 2.2.2 Data from PubMed

The search terms were: Mesh  =  (Sepsis) AND publication date  =  (1984/01/01–2014/12/31). Research from different countries was set as: affiliation  =  ((United States) OR (America)) or (Germany) or (France) or (UK) or (China). Refining: literature type  =  (randomized controlled trials) or (clinical trials) or (case reports). When searching for basic research, we refined species as “other Animals.”

### 2.3 Data collection

The text file data downloaded from WOS were imported into Microsoft Excel 2010, and then, 2 authors verified the data entry and collection. The final data were further manually analyzed in Microsoft Excel 2010. Bibliometric indicators, including the total publication number, total citation frequency, citation frequency per paper, h-index,^[14,15]^ research types, research orientations, research organization, author's contribution, journal and funding support were extracted from the original data to quantitatively and qualitatively analyze the publications.

### 2.4 Statistical methods

Graph Pad Prism 5.0 software was used for most of the statistical analyses. Linear regression was used to calculate the coefficient of determination (*r*^2^) and to determine any significant changes in trends between 1984 and 2014. *P* values (or Bonferroni corrected *P* values) less than .05 were considered significant.

## 3 Results

### 3.1 Global sepsis publication trends

#### 3.1.1 Amount of global sepsis articles and global growth trends

**Figure 1:**
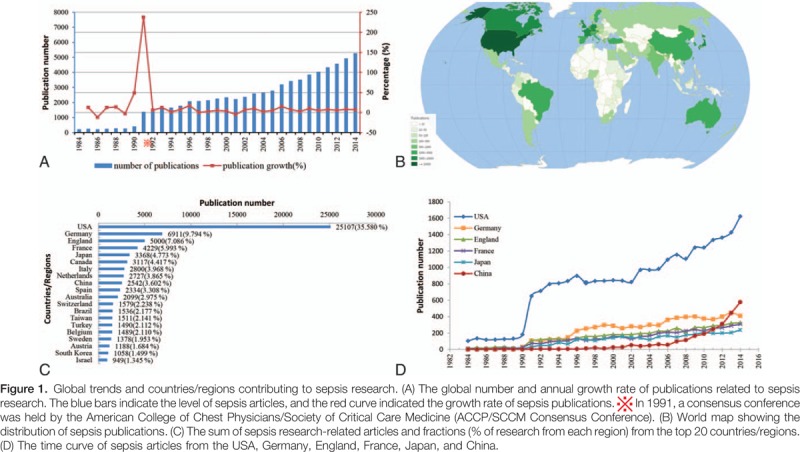


There were 70,564 sepsis articles that met the search criteria from 1984 to 2014. The global number of publications regarding sepsis showed a positive growth trend with an average annual growth rate of 11.11% (*r*^2^  =  0.96, *P* < .001), from 224 in 1984 to 5278 in 2014 (Fig. 1A, Online Supplementary Appendix 1A). A total of 177 countries and regions made contributions to the world's sepsis literature. Among these countries, the USA published the largest number of sepsis articles (25,107, 35.58%), followed by Germany (6911, 9.79%), England (5000, 7.09%), France (4229, 5.99%), and Japan (3368, 4.77%) (Fig. 1B and C, Online Supplementary Appendix 1B). The annual number of sepsis articles in the countries publishing the highest number of sepsis research papers all showed positive trends (Fig. 1D, Online Supplementary Appendix 1A).

#### 3.1.2 Citation and h-index analysis

**Figure 2:**
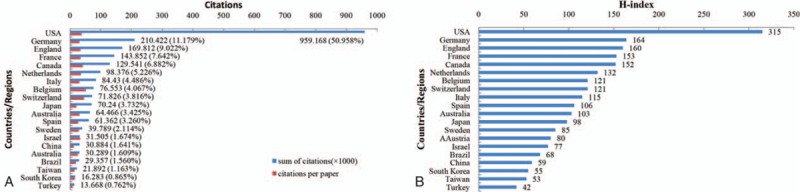
Citation frequency and h-index levels of different countries/regions. (A) The total citations, citation fraction (%), and citations per paper for sepsis articles from different countries/regions. (B) The h-index of sepsis publications in the different countries/regions.

According to the analysis of the WOS database, all articles related to sepsis had been cited 1,882,285 times since 1984 (1,811,154 times without self-citations) and the rate of self-citation is 3.8%. The cited frequency per paper was 26.67 times. Sepsis papers from the USA were cited most frequently, with 959,168 total citations (50.96%) and 38.203 citations per paper during the past 31 years (Fig. 2A, Online Supplementary Appendix 2). The h-index of papers published in the USA was 315, which was higher than that of any other country or region (Fig. 2B). Germany was the second largest contributor (210,422 citation frequency, 164 h-index), while England ranked third (169,812 citation frequency, 160 h-index) (Fig. 2A and B, Online Supplementary Appendix 2).

#### 3.1.3 The distribution of research types and research orientations

**Figure 3:**
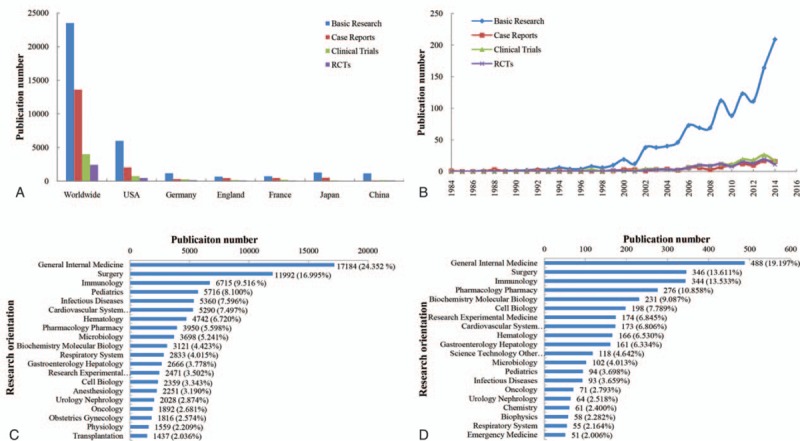
Article types and research orientations of Global Sepsis Research. (A) Comparison of published RCTs, clinical trials, case reports, and basic research studies for sepsis research amongst globally and in different countries. (B) Time curves for RCTs, clinical trials, case reports, and basic research in China between 1984 and 2014. (C) The sum of research orientations in the world. (D) The sum of research orientations in China. RCTs  =  randomized controlled trials.

Basic research was the main research types in the global sepsis research field with 23,521 papers, accounting for 29.20% of the total sepsis papers. Moreover, 13,625 case reports (16.92%), 4015 clinical trials (4.99%), and 2431 RCTs (3.02%) were published in the sepsis field. The number of publications for each research types from the USA was far ahead of the other countries (Fig. 3A, Online Supplementary Appendix 3A).

There were 110 research orientations related to global sepsis articles, among which general internal medicine (17,184, 24.35%), surgery (11,992, 17.00%), immunology (6715, 9.52%), pediatrics (5716, 8.10%), and infectious diseases (5360, 7.60%) were the most common areas (Fig. 3C).

#### 3.1.4 High contribution institutions/authors and main sepsis funding

**Figure 4:**
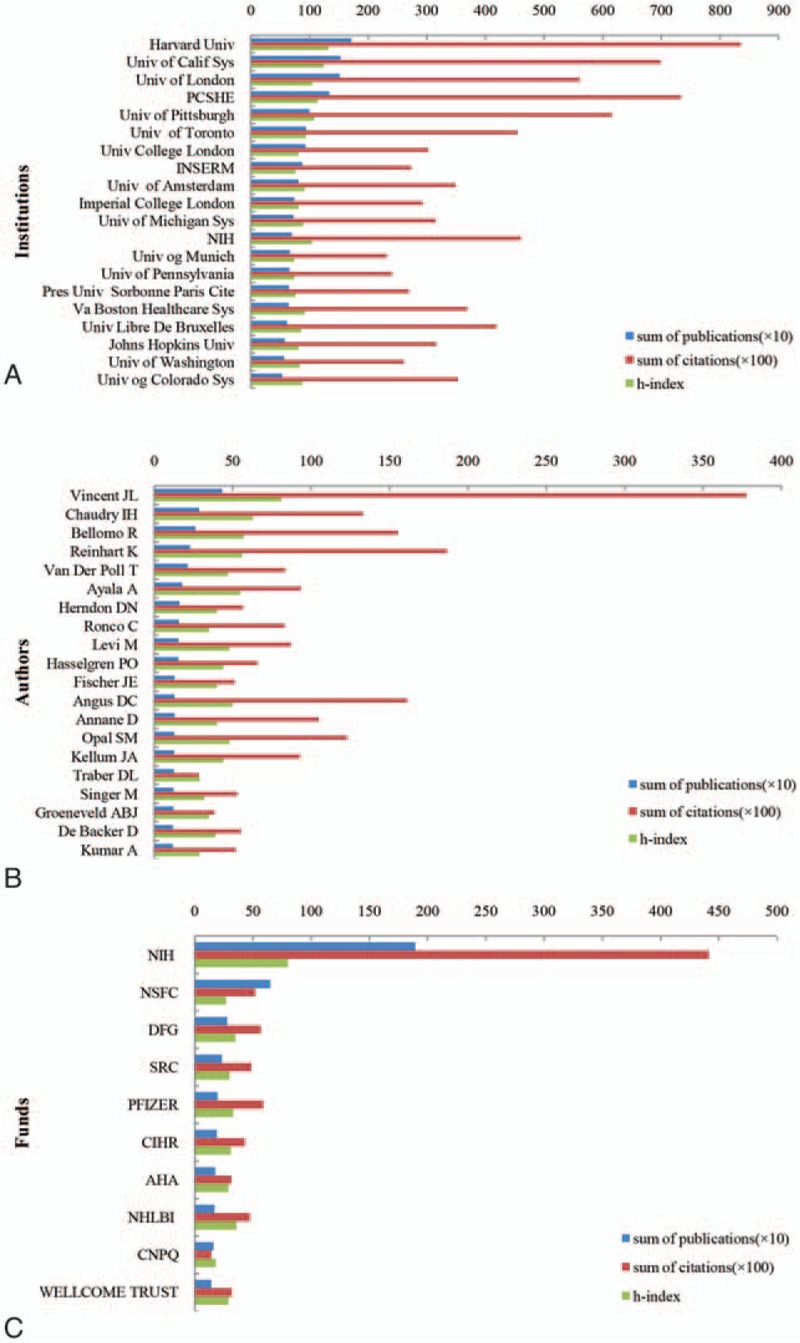
High impact institutions, authors, and funds of global sepsis research. (A) The high impact institutions in the world. (B) The high impact authors in the world. (C) The major contribution funds in the world. AHA  =  American Heart Association, CIHR  =  Canadian Institutes of Health Research, CNPQ  =  Conselho Nacional de Desenvolvimento Cientifico E Techologico, DFG  =  Deutsche Forschungsgemeinschaft, NHLBI  =  National Heart Lung and Blood Institute, NIH  =  National Institutes of Health, NSFC  =  National Natural Science Foundation of China, SRC  =  Swedish Research Council, Welcome Trust.

Twenty-six thousand five hundred three institutions from different nations or regions participated in sepsis research between 1984 and 2014. The top 20 most contributing institutions in the world are listed in Fig. 4A, which totaled 17,738 published sepsis articles accounting for 25.14%. Among these institutions, 11 were from the USA, 3 were from England, and 2 were from France. The institution with the largest amount of sepsis papers was Harvard University, which published 1716 papers and had the highest citation frequency (83,586) and h-index (132) as well (Fig. 4A, Online Supplementary Appendix 4A).

The top 20 most contributing authors published 3483 sepsis papers in total (4.94%). Among these 20 authors, 9 were from the USA, 3 were from the Netherlands, and 2 were from Belgium. Professor VINCENT JL, from Universite Libre Bruxelles was the most productive author and published 434 papers related to sepsis and had the highest citation frequency (37,774) and h-index (81) (Fig. 4B, Online Supplementary Appendix 4B).

Global sepsis articles were supported by 15,135 funding agencies. The top 10 most contributing funding agencies are listed in Fig. 4C. The National Institutes of Health (NIH) ranked first, which supported 1893 sepsis articles, with the highest citations (44,142) and h-index scores (80). NSFC was the second largest contributing funding agency, which supported 648 sepsis articles.

#### 3.1.5 High contribution journals

**Figure 5:**
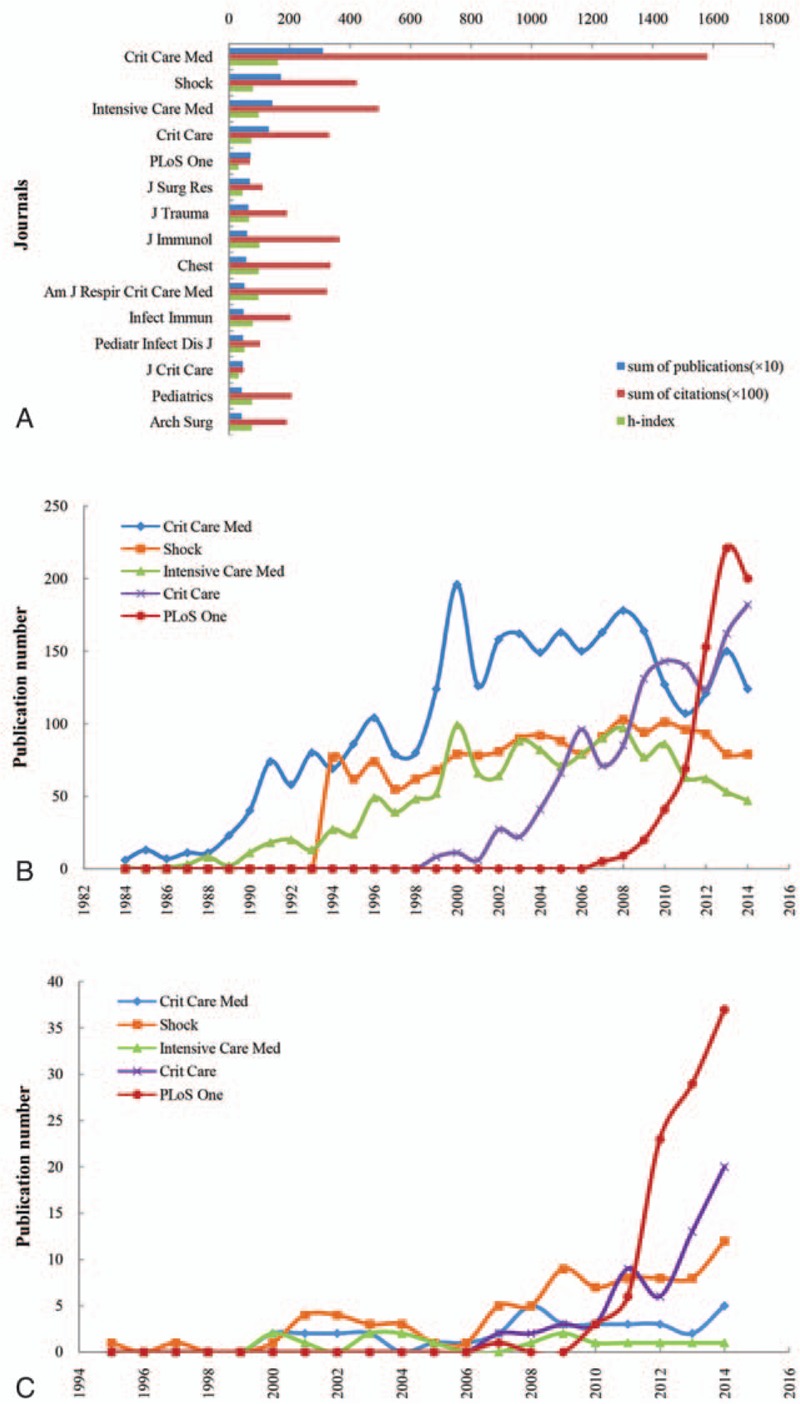
High contribution journals and growth trends of the main journals in sepsis field. (A) The main sepsis research journals in the world. (B) The time curves for Critical Care Medicine, Shock, Intensive Care Medicine, Critical Care, and PLoS One in the world. (C) The time curves for Critical Care Medicine, Shock, Intensive Care Medicine, Critical Care, and PLoS One in China.

A total of 3759 different journals published global sepsis articles from 1984 to 2014. Among these journals, 21 journals published more than 10 sepsis papers yearly, accounting for 22.29% (15,729/70,564) of the total number. Seventy-five journals published more than 5 papers yearly, accounting for 38.15% (26,920/70,564) of the total number. Two hundred twenty-nine journals published more than 2 papers yearly, accounting for 59.62% (42,072/70,564) of the total number. Among 3759 journals, Critical Care Medicine (IF 7.42) had the largest publication number (3103), the highest number of citations (158,11), and the highest h-index (161), followed by Shock (IF 3.05), Intensive Care Medicine (IF 10.13), Critical Care (IF 4.95), and PLoS One (IF 3.06) (Fig. 5A, Online Supplementary Appendix 5A).

The annual publication number of the above 5 most popular journals in the world was also searched, and they all showed a positive trend. PLoS One was the journal with the fastest increase in publications (from 5 papers in 2007 to 200 papers in 2014, average annual growth rate  =  69.381%, *r*^2^  =  0.88, *P* < .001) (Fig. 5B, Online Supplementary Appendix 5B).

### 3.2 Analysis of Chinese sepsis research

#### 3.2.1 China's growing trend of publications and contributions to global sepsis research

China, in total, published 2542 (3.60%) sepsis paper from 1984 to 2014. The number of published articles increased from 2 in 1984 to 576 in 2014. Meanwhile, annual amount of published articles from China showed a significant positive trend with an annual growth rate of 20.78% (*r*^2^  =  0.57, *P* < .001). China's publication numbers ranked third globally in 2012 (311) and second in 2014 (576) (Fig. 1C and D, Online Supplementary Appendix 1A and B).

#### 3.2.2 Citation and h-index analysis

The total citation frequency of China's sepsis articles was 30,884 (1.64%), ranking fifteenth in the world. In China, the citation frequency per paper was 12.15 and the h-index was 59 (Fig. 2A and B, Online Supplementary Appendix 2).

#### 3.2.3 Research types and research orientations in China

Basic research showed a clear dominant representation, with 1158 sepsis papers published between 1984 and 2014, accounting for 50.46% of China's sepsis articles (Fig. 3A, Online Supplementary Appendix 3A). One hundred forty clinical trials (6.10%), 107 RCTs (4.66%), and 103 case reports (4.49%) related to China's sepsis research were published. Positive growth trends were observed in all types, especially in basic research (average annual growth rate  =  21.88%, *r*^2^  =  0.77, *P* < .001) (Fig. 3B, Online Supplementary Appendix 3B).

There were 62 research areas found in China's sepsis articles. The main research orientations were general internal medicine (488, 19.20%), surgery (346, 13.61%), immunology (344, 13.53%), pharmacology pharmacy (276, 10.86%), and biochemistry molecular biology (231, 9.09%) (Fig. 3D).

#### 3.2.4 China's high contribution institutions and main sepsis funding

**Figure 6:**
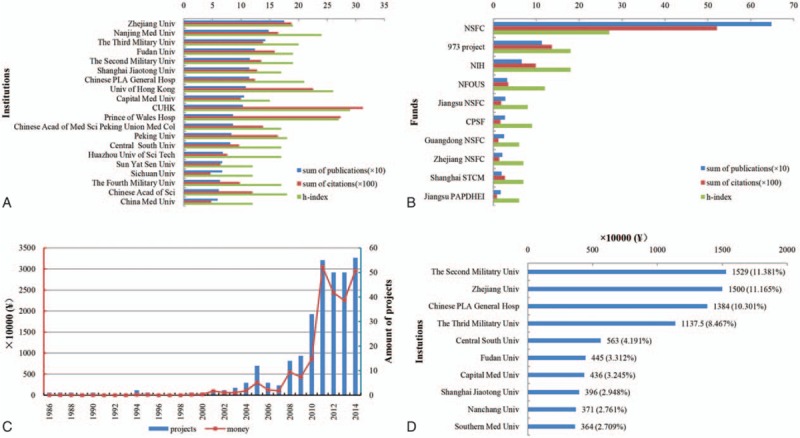
Contributing institutions, funds, and growth trends for NSFC of Sepsis Research in China. (A) The major institutions with sepsis research in China. (B) The main contribution funds in China. (C) The time curves for NSFC funding. The blue bars indicate the number of projects and the red curve indicates money. (D) NSFC funding of sepsis research at different institutions. 973 PROJECT  =  National Basic Research Program of China, CPSF  =  China Postdoctoral Science Foundation, Guangdong NSFC  =  National Natural Science Foundation of Guangdong Province China, Jiangsu NSFC  =  National Natural Science Foundation of Jiangsu Province China, Jiangsu PAPDHEI  =  Priority Academic Program Development of Jiangsu Higher Education Institutions, NIH  =  National Institutes of Health, NNFOYS  =  National Funds for Outstanding Youth Scientists, NSFC  =  National Natural Science Foundation of China, Shanghai STCM  =  Science and Technology Commission of Shanghai Municipality, Zhejiang NSFC  =  National Natural Science Foundation of Zhejiang Province China.

A total of 1762 institutions participated in sepsis research between 1984 and 2014 in China. The top 20 contributing institutions published 1969 sepsis articles in total, accounting for 77.46%. These research centers were mainly located in Beijing (5), Shanghai (4), and Hong Kong (3). Zhejiang University, with 175 sepsis papers published, was the largest research institution. Chinese University of Hong Kong (CUHK) had the highest citation (3123) and h-index (29) rates (Fig. 6A, Online Supplementary Appendix 6A).

The top 10 funding agencies that supported China's sepsis research are listed in Fig. 6B. NSFC supported the most Chinese sepsis articles (648, 25.49%), followed by 973 project (113, 4.45%). The NSFC was the main Chinese funding organization. The fund supplied 134.35 million RMB for sepsis research from 1986 to 2014, with an average annual growth rate 29.78% (*r*^2^  =  0.48, *P* < .001) (Fig. 6C, Online Supplementary Appendix 6B). The Second Military Medical University gained the largest amount of NSFC funding (15.29 million RMB), followed by Zhejiang University (15 million) and Chinese People's Liberation Army General Hospital (13.84 million) (Fig. 6D).

#### 3.2.5 Popular journals in China

The annual number of publications in the top 5 most popular global journals from Chinese authors showed a positive growth trend. The number of papers in PLoS One from China increased most rapidly, with an average annual growth rate  =  67.51% (*r*^2^  =  0.84, *P*  =  .001) (Fig. 5C, Online Supplementary Appendix 5C). Chinese Medical Journal (IF 0.96) was most popular in China, with 110 published sepsis articles, accounting for 4.33% of the total, which was followed by PLoS One (IF 3.06) (99, 3.90%) and Shock (IF 3.05) (81, 3.19%) (Online Supplementary Appendix 7).

## 4 Discussion

Our present bibliometric study provided a comprehensive overview of the development of the scientific literature in global sepsis research and summarized the contribution of China over the last 3 decades by comparing the quality and quantity in sepsis research articles. Worldwide sepsis research had made a tremendous increase in volume in the last 3 decades. The USA led this field with the highest publications, citations, and h-index. Sepsis research in China had a notable trend of increase in a number of scientific publications. However, the quality of China's sepsis research remained relatively lower when compared with the fast-growing quantities. In subcategories of research, researchers had relatively higher output in basic research than in clinical investigations. The NIH contributed the most funding to global sepsis research, while the NSFC was the most dominating funder in China.

Great advances were observed in global sepsis research, with 224 publications in 1984 increasing to 5278 in 2014, especially after the first Consensus Conference for Sepsis Definition held by ACCP/SCCM in 1991.^[4]^ This Consensus Conference firstly proposed the terms of sepsis, severe sepsis, and septic shock, and put forward specific definitions for them, which marked a new era for this field with an unprecedented growth rate of 273.41% in 1991. Thus, successfully hosting this conference in 1991 may be the underlying reason why there was a substantial increase in the publications on sepsis between 1990 and 1992. Another on-going positive growth in sepsis literature occurred after 2003 when the Surviving Sepsis Campaign launched its evidence-based guideline to improve sepsis outcomes.^[16]^ In 2016, the third international consensus conference for sepsis and septic shock definitions provided new definition and diagnostic criteria for sepsis, which may cause great repercussions to sepsis research in the future.^[2]^

Sepsis research in China developed slowly in the early stage, but erupted after 2001, reaching 576 articles in 2014, which ranked second in the world. The recent fast development of sepsis research in China can be mainly ascribed to: The unprecedented growth in their economy in the last 3 decades in China, which pushed sepsis research forward. Many bibliometric studies have confirmed the significant roles of the economy, science, and technology in the progress of other disciplines.^[8–10,12,13]^ China has increased its scientific research investments year-by-year. Additionally, increased governmental funding agencies, such as the NSFC and the 973 project, have enhanced research productivity. Many Chinese experts come back with well-recognized experience, advanced techniques and bring more international collaborations in sepsis diagnoses and therapies. The large population base in China is associated with a large number of patients to study, which may bring more resources to sepsis studies.

However, there is still a large gap between China and other leading countries in terms of the citation frequency and h-index. The relatively lower quality of sepsis publications from China may be due to: China lacks highly influential leading researchers and research institutions to implement high quality sepsis research. In our analysis, there were no Chinese researchers or institutions that ranked in the global top 20 contributing authors and institutions. The immaturity of the research system and the lack of experience in high quality research may be caused by the relatively later initiation of sepsis research in China. Second, China also lacks top international journals. The most popular journal that published China's sepsis research was the Chinese Medical Journal (IF 0.957), which has much less impact than other sepsis related journals. Third, the journals with high IF are usually English-written, while for China, as a non-English-speaking country, there may difficulty in writing scientific papers in English.

The present study indicated that researchers in China have relatively better output in basic research than in clinical investigation, This could be mainly attributed to the rapid development of NSFC during the past decade, which has the widest coverage in China and principally supports basic scientific researches.^[17]^ On the other hand, China lacks clinical cooperating platforms and well-developed big database and analysis system. The vast amount of clinical information and research resources in different hospitals are relatively independent, resulting in a difficulty in carrying out multicenters clinical studies. Nevertheless, clinical study of sepsis in China still has great potential, as it is less expensive to perform clinical trials in China than in the developed countries and easy to recruit a large number of subjects.

There are some limitations in our study. First, we used the terms “sepsis” or “septic shock” in the topic field, which may have caused us to not identify some publications, such as those indexed with “Inflammation.” Second, delayed publication collections from the WOS and PubMed databases can also cause bias in the study. Some papers may have already published in SCI journals, but have not been indexed by the above 2 databases. Third, the WOS and PubMed databases mainly included English-written pieces of literatures. Many non-English publications and publications in some databases or university libraries were not included.

## 5 Conclusion

Sepsis research in the world had made tremendous grown in volume during the last 3 decades, especially after the first Consensus Conference held by ACCP and SCCM in 1991. The USA leads the development of sepsis research with the most publications, highest citations, and h-index. Both the quantity and the quality of publications in sepsis research in China were remarkably improved over the past 31 years, and China became the second largest contributor by 2014. However, compared with the enormous growth in the quantity of publications, there is still a large gap between China and other leading countries in the term of research quality. Therefore, it is quite necessary to take steps to conduct high-quality sepsis studies in China.

## Supplementary Material

Supplemental Digital Content
