# Supplementary material for: China's growing contribution to sepsis research from 1984 to 2014: A bibliometric study
Source: Medicine (Baltimore). 2017 Jun 23;96(25):e7275. doi: 10.1097/MD.0000000000007275 (PMC5484245; doi:10.1097/MD.0000000000007275)
Supplement: Supplemental Digital Content [file medi-96-e7275-s001.docx]

Supplementary Appendix 1. Global trends and countries/regions contributing to sepsis research.

A. Annual number of sepsis articles from the five leading countries and China between 1984 and 2014.

| Year | Global | USA | Germany | England | France | Japan | China |
| --- | --- | --- | --- | --- | --- | --- | --- |
| 1984 | 224 | 104 | 0 | 12 | 10 | 2 | 2 |
| 1985 | 252 | 134 | 0 | 20 | 5 | 0 | 0 |
| 1986 | 222 | 118 | 0 | 16 | 7 | 1 | 1 |
| 1987 | 249 | 120 | 0 | 26 | 8 | 1 | 1 |
| 1988 | 283 | 126 | 0 | 28 | 19 | 2 | 1 |
| 1989 | 275 | 131 | 1 | 19 | 13 | 5 | 2 |
| 1990 | 409 | 182 | 28 | 29 | 27 | 9 | 0 |
| 1991 | 1380 | 650 | 107 | 107 | 71 | 32 | 5 |
| 1992 | 1457 | 713 | 103 | 118 | 71 | 48 | 6 |
| 1993 | 1636 | 797 | 120 | 128 | 94 | 50 | 6 |
| 1994 | 1659 | 807 | 123 | 129 | 101 | 83 | 4 |
| 1995 | 1783 | 834 | 147 | 118 | 111 | 68 | 6 |
| 1996 | 2090 | 897 | 226 | 136 | 131 | 120 | 6 |
| 1997 | 2094 | 815 | 254 | 159 | 110 | 124 | 12 |
| 1998 | 2144 | 834 | 271 | 160 | 127 | 109 | 20 |
| 1999 | 2256 | 837 | 297 | 170 | 132 | 130 | 12 |
| 2000 | 2339 | 843 | 288 | 183 | 158 | 142 | 26 |
| 2001 | 2224 | 838 | 257 | 180 | 155 | 157 | 28 |
| 2002 | 2375 | 822 | 280 | 184 | 127 | 145 | 53 |
| 2003 | 2599 | 970 | 272 | 195 | 157 | 140 | 42 |
| 2004 | 2657 | 970 | 299 | 199 | 166 | 120 | 48 |
| 2005 | 2785 | 981 | 296 | 223 | 198 | 156 | 62 |
| 2006 | 3195 | 1094 | 361 | 223 | 210 | 166 | 56 |
| 2007 | 3427 | 1155 | 386 | 258 | 207 | 151 | 98 |
| 2008 | 3519 | 1105 | 396 | 219 | 225 | 175 | 114 |
| 2009 | 3859 | 1242 | 401 | 267 | 240 | 185 | 165 |
| 2010 | 4044 | 1242 | 376 | 267 | 229 | 206 | 190 |
| 2011 | 4339 | 1334 | 368 | 288 | 257 | 197 | 245 |
| 2012 | 4577 | 1364 | 400 | 294 | 265 | 200 | 311 |
| 2013 | 4934 | 1426 | 445 | 319 | 289 | 206 | 444 |
| 2014 | 5278 | 1622 | 409 | 326 | 309 | 238 | 576 |
| Total | 70564 | 25107 | 6911 | 5000 | 4229 | 3368 | 2542 |
| Average Annual Growth Rate (%) | 11.107 | 9.589 | 27.195 | 11.635 | 12.115 | 17.269 | 20.776 |
| R- Square | 0.9614 | 0.9017 | 0.9084 | 0.9661 | 0.9724 | 0.9463 | 0.5650 |
| P-Value | < 0.0001 | < 0.0001 | < 0.0001 | < 0.0001 | < 0.0001 | < 0.0001 | < 0.0001 |

B. The sum of sepsis research-related articles and fractions (percentage of research from each country/region) from different countries/regions between 1984 and 2014.

| Country | Publications | Percentage |
| --- | --- | --- |
| USA | 25107 | 35.58 |
| Germany | 6911 | 9.794 |
| England | 5000 | 7.086 |
| France | 4229 | 5.993 |
| Japan | 3368 | 4.773 |
| Canada | 3117 | 4.417 |
| Italy | 2800 | 3.968 |
| Netherlands | 2727 | 3.865 |
| China | 2542 | 3.602 |
| Spain | 2334 | 3.308 |
| Australia | 2099 | 2.975 |
| Switzerland | 1579 | 2.238 |
| Brazil | 1536 | 2.177 |
| Taiwan | 1511 | 2.141 |
| Turkey | 1490 | 2.112 |
| Belgium | 1489 | 2.11 |
| Sweden | 1378 | 1.953 |
| Austria | 1188 | 1.684 |
| South Korea | 1058 | 1.499 |
| Israel | 949 | 1.345 |
| India | 944 | 1.338 |
| Greece | 803 | 1.138 |
| Scotland | 729 | 1.033 |
| Denmark | 621 | 0.88 |
| Finland | 494 | 0.7 |
| Poland | 438 | 0.621 |
| South Africa | 436 | 0.618 |
| Norway | 418 | 0.592 |
| Ireland | 376 | 0.533 |
| Argentina | 336 | 0.476 |
| Singapore | 313 | 0.444 |
| Saudi Arabia | 311 | 0.441 |
| New Zealand | 311 | 0.441 |
| Czech Republic | 293 | 0.415 |
| Hungary | 275 | 0.39 |
| Mexico | 266 | 0.377 |
| Chile | 262 | 0.371 |
| Thailand | 243 | 0.344 |
| Iran | 213 | 0.302 |
| Russia | 200 | 0.283 |
| Egypt | 192 | 0.272 |
| Portugal | 188 | 0.266 |
| Wales | 176 | 0.249 |
| Croatia | 162 | 0.23 |
| Pakistan | 159 | 0.225 |
| Nigeria | 150 | 0.213 |
| North Ireland | 146 | 0.207 |
| Ussr | 113 | 0.16 |
| Serbia | 99 | 0.14 |
| Malaysia | 95 | 0.135 |
| Tunisia | 94 | 0.133 |
| Colombia | 89 | 0.126 |
| Romania | 86 | 0.122 |
| Slovenia | 85 | 0.12 |
| Kenya | 70 | 0.099 |
| Bangladesh | 69 | 0.098 |
| Hong Kong | 68 | 0.096 |
| Fed Rep Ger | 68 | 0.096 |
| Kuwait | 62 | 0.088 |
| Slovakia | 57 | 0.081 |
| Morocco | 53 | 0.075 |
| Uganda | 48 | 0.068 |
| Malawi | 48 | 0.068 |
| Indonesia | 47 | 0.067 |
| Lebanon | 44 | 0.062 |
| Cuba | 44 | 0.062 |
| Uruguay | 42 | 0.06 |
| Nepal | 42 | 0.06 |
| Estonia | 42 | 0.06 |
| Lithuania | 41 | 0.058 |
| U Arab Emirates | 38 | 0.054 |
| Bulgaria | 38 | 0.054 |
| Philippines | 36 | 0.051 |
| Iceland | 36 | 0.051 |
| Ethiopia | 36 | 0.051 |
| Jamaica | 34 | 0.048 |
| Vietnam | 33 | 0.047 |
| Venezuela | 33 | 0.047 |
| Tanzania | 33 | 0.047 |
| Ghana | 32 | 0.045 |
| Papua N Guinea | 27 | 0.038 |
| Jordan | 27 | 0.038 |
| Qatar | 26 | 0.037 |
| Zimbabwe | 23 | 0.033 |
| Yugoslavia | 19 | 0.027 |
| Trinid Tobago | 19 | 0.027 |
| Ukraine | 16 | 0.023 |
| Peru | 16 | 0.023 |
| Gambia | 16 | 0.023 |
| Zambia | 15 | 0.021 |
| Sri Lanka | 15 | 0.021 |
| Senegal | 15 | 0.021 |
| Cameroon | 15 | 0.021 |
| Byelarus | 15 | 0.021 |
| Sudan | 14 | 0.02 |
| Reunion | 14 | 0.02 |
| Bosnia Herceg | 14 | 0.02 |
| Oman | 13 | 0.018 |
| Latvia | 13 | 0.018 |
| Costa Rica | 13 | 0.018 |
| Iraq | 12 | 0.017 |
| Luxembourg | 11 | 0.016 |
| Gabon | 11 | 0.016 |
| Ecuador | 11 | 0.016 |
| Guatemala | 10 | 0.014 |
| Bahrain | 10 | 0.014 |
| Panama | 9 | 0.013 |
| New Caledonia | 9 | 0.013 |
| Monaco | 9 | 0.013 |
| Mozambique | 8 | 0.011 |
| Mali | 8 | 0.011 |
| Libya | 8 | 0.011 |
| Laos | 8 | 0.011 |
| Ger Dem Rep | 8 | 0.011 |
| Cote Ivoire | 8 | 0.011 |
| Brunei | 8 | 0.011 |
| Neth Antilles | 7 | 0.01 |
| Mongol Peo Rep | 7 | 0.01 |
| Guadeloupe | 7 | 0.01 |
| Rep of Georgia | 6 | 0.009 |
| Zaire | 5 | 0.007 |
| W Ind Assoc St | 5 | 0.007 |
| Niger | 5 | 0.007 |
| Malta | 5 | 0.007 |
| Guinea Bissau | 5 | 0.007 |
| Grenada | 5 | 0.007 |
| Burkina Faso | 5 | 0.007 |
| Bolivia | 5 | 0.007 |
| Togo | 4 | 0.006 |
| Paraguay | 4 | 0.006 |
| Kazakhstan | 4 | 0.006 |
| Guinea | 4 | 0.006 |
| French Guiana | 4 | 0.006 |
| Cyprus | 4 | 0.006 |
| Congo | 4 | 0.006 |
| Benin | 4 | 0.006 |
| Armenia | 4 | 0.006 |
| Algeria | 4 | 0.006 |
| Albania | 4 | 0.006 |
| Syria | 3 | 0.004 |
| Serbia Monteneg | 3 | 0.004 |
| Nicaragua | 3 | 0.004 |
| Montenegro | 3 | 0.004 |
| Haiti | 3 | 0.004 |
| Cambodia | 3 | 0.004 |
| Vanuatu | 2 | 0.003 |
| Rwanda | 2 | 0.003 |
| Myanmar | 2 | 0.003 |
| Madagascar | 2 | 0.003 |
| Macedonia | 2 | 0.003 |
| Honduras | 2 | 0.003 |
| Fiji | 2 | 0.003 |
| Eritrea | 2 | 0.003 |
| Dominica | 2 | 0.003 |
| Angola | 2 | 0.003 |
| Afghanistan | 2 | 0.003 |
| Yemen | 1 | 0.001 |
| Vatican | 1 | 0.001 |
| Uzbekistan | 1 | 0.001 |
| Ukssr | 1 | 0.001 |
| Swaziland | 1 | 0.001 |
| Sierra Leone | 1 | 0.001 |
| Senegambia | 1 | 0.001 |
| Namibia | 1 | 0.001 |
| Moldova | 1 | 0.001 |
| Martinique | 1 | 0.001 |
| Jersey | 1 | 0.001 |
| Guyana | 1 | 0.001 |
| El Salvador | 1 | 0.001 |
| Cook Islands | 1 | 0.001 |
| Ciskei | 1 | 0.001 |
| Cent Afr Republ | 1 | 0.001 |
| Botswana | 1 | 0.001 |
| Bessr | 1 | 0.001 |
| Bermuda | 1 | 0.001 |
| Barbados | 1 | 0.001 |
| Antigua Barbu | 1 | 0.001 |

Supplementary Appendix 2. The annual citation frequency related to sepsis articles from the five leading countries and China during 1984 to 2014.

| Year | Global | USA | Germany | England | France | Japan | China |
| --- | --- | --- | --- | --- | --- | --- | --- |
| 1984 | 35 | 22 | 0 | 3 | 0 | 0 | 0 |
| 1985 | 370 | 235 | 0 | 20 | 11 | 0 | 0 |
| 1986 | 1034 | 656 | 0 | 60 | 35 | 0 | 0 |
| 1987 | 2182 | 1430 | 0 | 123 | 62 | 0 | 0 |
| 1988 | 3926 | 2593 | 0 | 238 | 96 | 4 | 0 |
| 1989 | 5934 | 3956 | 0 | 363 | 125 | 10 | 0 |
| 1990 | 8689 | 5784 | 1 | 548 | 199 | 22 | 5 |
| 1991 | 12381 | 8264 | 40 | 779 | 279 | 27 | 9 |
| 1992 | 18397 | 12311 | 187 | 1171 | 435 | 71 | 15 |
| 1993 | 28782 | 19133 | 625 | 1944 | 725 | 184 | 29 |
| 1994 | 43296 | 28561 | 1415 | 3102 | 1220 | 424 | 43 |
| 1995 | 63507 | 41192 | 2811 | 4800 | 2034 | 877 | 68 |
| 1996 | 92542 | 58512 | 4893 | 7294 | 3345 | 1641 | 106 |
| 1997 | 123946 | 77095 | 7420 | 9974 | 4951 | 2603 | 144 |
| 1998 | 161299 | 98817 | 10852 | 13124 | 7019 | 3933 | 187 |
| 1999 | 203650 | 122791 | 15132 | 16710 | 9444 | 5425 | 246 |
| 2000 | 252123 | 149966 | 20400 | 20628 | 12367 | 7326 | 312 |
| 2001 | 305285 | 178751 | 26576 | 25015 | 15739 | 9471 | 449 |
| 2002 | 361814 | 209383 | 33255 | 29710 | 19587 | 11967 | 655 |
| 2003 | 425479 | 243506 | 40827 | 35050 | 24491 | 14742 | 923 |
| 2004 | 494213 | 280072 | 48768 | 40718 | 29700 | 17591 | 1281 |
| 2005 | 569631 | 319247 | 57339 | 47101 | 35487 | 20622 | 1820 |
| 2006 | 654839 | 363083 | 67050 | 54391 | 42318 | 24090 | 2559 |
| 2007 | 748496 | 410920 | 77856 | 62441 | 49784 | 27660 | 3546 |
| 2008 | 852374 | 463063 | 90054 | 71443 | 58271 | 31485 | 4767 |
| 2009 | 965916 | 520121 | 103559 | 81797 | 67693 | 35503 | 6164 |
| 2010 | 1089829 | 581313 | 118385 | 93151 | 77995 | 40135 | 8114 |
| 2011 | 1226951 | 648127 | 134301 | 106107 | 89460 | 45348 | 10621 |
| 2012 | 1372439 | 719139 | 151111 | 119973 | 101188 | 50849 | 13692 |
| 2013 | 1530088 | 793844 | 169619 | 135437 | 114273 | 56802 | 17941 |
| 2014 | 1697409 | 873210 | 189124 | 151694 | 128301 | 63214 | 23565 |
| 2015 | 1868893 | 952687 | 209057 | 168613 | 142748 | 69736 | 30331 |
| 2016 | 1882285 | 959168 | 210422 | 169812 | 143852 | 70240 | 30884 |
| Average Annual Growth Rate (%) | 43.776 | 42.774 | 66.646 | 44.022 | 38.659 | 45.630 | 43.863 |
| R- Square | 0.8378 | 0.8598 | 0.8083 | 0.8191 | 0.7876 | 0.8212 | 0.5608 |
| P-Value | < 0.0001 | < 0.0001 | < 0.0001 | < 0.0001 | < 0.0001 | < 0.0001 | < 0.0001 |

Supplementary Appendix 3. Global and China’s research types.

A. Comparison of published articles of RCTs, clinical trials, case reports and basic researches of sepsis research among the world and different countries.

| Research Types | Global (n=80654) | USA (n=15486) | Germany (n=3131) | England (n=2925) | France (n=2942) | Japan (n=2756) | China (n=2295) |
| --- | --- | --- | --- | --- | --- | --- | --- |
| RCTs, n (%) | 2431 (3.018) | 471 (3.041) | 144 (4.599) | 104 (3.556) | 98 (3.331) | 30 (1.089) | 107 (4.662) |
| Clinical Trials, n (%) | 4015 (4.985) | 744 (4.804) | 268 (8.560) | 156 (5.333) | 210 (7.138) | 105 (3.810) | 140 (6.100) |
| Case Reports, n (%) | 13625 (16.917) | 2042 (13.186) | 314 (10.029) | 463 (15.829) | 474 (16.111) | 518 (18.795) | 103 (4.488) |
| Basic Research, n (%) | 23521 (29.203) | 5984 (38.641) | 1171 (37.400) | 667 (22.803) | 732 (24.881) | 1273 (46.190) | 1158 (50.458) |

RCTs: randomized controlled trials.

B. The annual sepsis articles of RCTs, clinical trials, case reports and basic research in China between 1984 and 2014.

| Year | Basic Research | Case Reports | Clinical Trials | RCTs |
| --- | --- | --- | --- | --- |
| 1984 | 0 | 1 | 0 | 0 |
| 1985 | 0 | 0 | 0 | 0 |
| 1986 | 0 | 0 | 0 | 0 |
| 1987 | 1 | 0 | 0 | 0 |
| 1988 | 1 | 3 | 0 | 0 |
| 1989 | 1 | 0 | 0 | 0 |
| 1990 | 1 | 0 | 0 | 0 |
| 1991 | 2 | 0 | 0 | 0 |
| 1992 | 3 | 2 | 0 | 0 |
| 1993 | 3 | 0 | 0 | 0 |
| 1994 | 6 | 1 | 0 | 0 |
| 1995 | 4 | 0 | 0 | 0 |
| 1996 | 4 | 0 | 0 | 0 |
| 1997 | 8 | 1 | 3 | 0 |
| 1998 | 6 | 0 | 0 | 0 |
| 1999 | 10 | 1 | 2 | 2 |
| 2000 | 19 | 3 | 1 | 1 |
| 2001 | 12 | 3 | 1 | 0 |
| 2002 | 38 | 1 | 4 | 2 |
| 2003 | 38 | 3 | 4 | 3 |
| 2004 | 40 | 4 | 3 | 3 |
| 2005 | 46 | 2 | 3 | 3 |
| 2006 | 73 | 6 | 7 | 6 |
| 2007 | 69 | 6 | 10 | 10 |
| 2008 | 69 | 3 | 9 | 9 |
| 2009 | 112 | 7 | 12 | 12 |
| 2010 | 88 | 10 | 11 | 8 |
| 2011 | 123 | 12 | 19 | 15 |
| 2012 | 111 | 10 | 18 | 13 |
| 2013 | 164 | 17 | 26 | 19 |
| 2014 | 209 | 16 | 17 | 11 |
| Average Annual Growth Rate (%) | 21.880 | 9.356 | 10.742 | 12.036 |
| R- Square | 0.7675 | 0.6370 | 0.8132 | 0.8161 |
| P-Value | < 0.0001 | < 0.0001 | < 0.0001 | < 0.0001 |

RCTs: randomized controlled trials.

Supplementary Appendix 4. Global high impact institutions and authors.

A. Number of articles, citations and h-index from top 20 highest impact institutions in the world.

| Institutions | publications | Percentage (n=70564) | Citation Frequency | H-index | Countries/Regions |
| --- | --- | --- | --- | --- | --- |
| Harvard University | 1716 | 2.432 | 83586 | 132 | USA |
| University of California System | 1530 | 2.168 | 69881 | 124 | USA |
| University of London | 1518 | 2.151 | 56015 | 105 | England |
| Pennsylvania Commonwealth System of Higher Education Pcshe | 1338 | 1.896 | 73328 | 114 | USA |
| University of Pittsburgh | 1002 | 1.42 | 61589 | 108 | USA |
| University of Toronto | 941 | 1.334 | 45430 | 94 | Canada |
| University College London | 928 | 1.315 | 30293 | 81 | England |
| Institut National De La Sante Et De La Recherche Medicale Inserm | 874 | 1.239 | 27346 | 76 | France |
| University of Amsterdam | 810 | 1.148 | 34922 | 92 | Netherlands |
| Imperial College London | 739 | 1.047 | 29291 | 81 | England |
| University of Michigan | 727 | 1.03 | 31533 | 89 | USA |
| National Institutes of Health NIH USA | 699 | 0.991 | 46013 | 104 | USA |
| University of Munich | 667 | 0.945 | 23206 | 74 | Germany |
| University of Pennsylvania | 657 | 0.931 | 24066 | 74 | USA |
| Pres University Sorbonne Paris Cite | 651 | 0.923 | 26999 | 76 | France |
| Va Boston Healthcare System | 646 | 0.915 | 36864 | 92 | USA |
| Universite Libre De Bruxelles | 619 | 0.877 | 41834 | 86 | Belgium. |
| Johns Hopkins University | 578 | 0.819 | 31653 | 81 | USA |
| University of Washington | 567 | 0.804 | 26096 | 84 | USA |
| University of Colorado System | 531 | 0.753 | 35309 | 88 | USA |
| Total | 17738 | 25.138 | 835254 | / | / |

B. Number of articles, citations and h-index from top 20 highest impact authors in the world.

| Authors | Publications | Percentage (n=70564) | Citation Frequency | H-index | Organization | Countries/Regions |
| --- | --- | --- | --- | --- | --- | --- |
| Vincent JL | 434 | 0.615 | 37774 | 81 | Universite Libre de Bruxelles | Belgium. |
| Chaudry IH | 287 | 0.407 | 13315 | 63 | University of Alabama Birmingham or  University of Alabama System | USA |
| Bellomo R | 262 | 0.371 | 15535 | 57 | Monash University/(Austin Research Institute and Howard Florey Institute) | Australia |
| Reinhart K | 228 | 0.323 | 18668 | 56 | Friedrich Schiller University of Jena | Germany |
| Van Der Poll T | 213 | 0.302 | 8359 | 47 | University of Amsterdam | Netherlands |
| Ayala A | 178 | 0.252 | 9347 | 55 | Brown University | USA |
| Herndon DN | 162 | 0.23 | 5658 | 40 | University of Texas Medical Branch Galveston | USA |
| Ronco C | 157 | 0.222 | 8313 | 35 | San Bortolo Hosp | Italy |
| Levi M | 155 | 0.22 | 8719 | 48 | University of Amsterdam | Netherlands |
| Hasselgren  PO | 153 | 0.217 | 6594 | 44 | Harvard University | USA |
| Fischer JE | 130 | 0.184 | 5132 | 40 | Harvard University | USA |
| Angus DC | 130 | 0.184 | 16120 | 50 | University of Pittsburgh | USA |
| Annane D | 129 | 0.183 | 10504 | 40 | University of Versailles Saint Quentin En Yvelines or Hop Raymond Poincare | France |
| Opal SM | 128 | 0.181 | 12307 | 48 | Brown University | USA |
| Kellum JA | 127 | 0.18 | 9302 | 44 | University of Pittsburgh | USA |
| Traber DL | 126 | 0.179 | 2846 | 29 | University of Texas Medical Branch Galveston | USA |
| Singer M | 123 | 0.174 | 5313 | 32 | University College London | England |
| Groeneveld ABJ | 122 | 0.173 | 3847 | 35 | Erasmus University Rotterdam | Netherlands |
| De Backer D | 120 | 0.17 | 5529 | 39 | Universite Libre de Bruxelles | Belgium |
| Kumar A | 119 | 0.169 | 5203 | 29 | University of Manitoba | Canada |
| Total | 3483 | 4.936 | 208385 | / | / | / |

Supplementary Appendix 5. Global journals published sepsis articles and growth trend of the most popular journals in the world and in China.

1. Global journals that published more than 10,5 and 2 sepsis papers per year during 1984-2014.

| Journals | Publications | Percentage (n=70564) |
| --- | --- | --- |
| Critical Care Medicine | 3103 | 4.397 |
| Shock | 1721 | 2.439 |
| Intensive Care Medicine | 1440 | 2.041 |
| Critical Care | 1315 | 1.864 |
| PLoS One | 718 | 1.018 |
| Journal of Surgical Research | 690 | 0.978 |
| Journal of Trauma Injury Infection And Critical Care | 636 | 0.901 |
| Journal of Immunology | 600 | 0.85 |
| Chest | 567 | 0.804 |
| American Journal of Respiratory And Critical Care Medicine | 508 | 0.72 |
| Infection And Immunity | 482 | 0.683 |
| Pediatric Infectious Disease Journal | 464 | 0.658 |
| Journal of Critical Care | 460 | 0.652 |
| Pediatrics | 418 | 0.592 |
| Archives of Surgery | 416 | 0.59 |
| Journal of Infectious Diseases | 404 | 0.573 |
| Clinical Infectious Diseases | 403 | 0.571 |
| Surgery | 359 | 0.509 |
| Journal of Pediatric Surgery | 359 | 0.509 |
| Annals of Surgery | 346 | 0.49 |
| British Journal of Surgery | 320 | 0.453 |
| >=10 per year (21 journals) | 15729 | 22.290 |
| American Journal of Surgery | 287 | 0.407 |
| Journal of Clinical Microbiology | 286 | 0.405 |
| Burns | 286 | 0.405 |
| Transplantation Proceedings | 285 | 0.404 |
| New England Journal of Medicine | 283 | 0.401 |
| Blood | 277 | 0.393 |
| Acta Anaesthesiologica Scandinavica | 270 | 0.383 |
| Circulatory Shock | 259 | 0.367 |
| Journal of Pediatrics | 253 | 0.359 |
| American Surgeon | 246 | 0.349 |
| Lancet | 243 | 0.344 |
| Journal of Biological Chemistry | 240 | 0.34 |
| Journal of Antimicrobial Chemotherapy | 238 | 0.337 |
| Cytokine | 236 | 0.334 |
| Journal of Parenteral And Enteral Nutrition | 233 | 0.33 |
| Pediatric Critical Care Medicine | 232 | 0.329 |
| Journal of Leukocyte Biology | 232 | 0.329 |
| Scandinavian Journal of Infectious Diseases | 229 | 0.325 |
| Jama Journal of The American Medical Association | 226 | 0.32 |
| Diseases of The Colon Rectum | 221 | 0.313 |
| Antimicrobial Agents And Chemotherapy | 219 | 0.31 |
| Acta Paediatrica | 214 | 0.303 |
| World Journal of Surgery | 212 | 0.3 |
| European Journal of Clinical Microbiology Infectious Diseases | 210 | 0.298 |
| Anesthesia And Analgesia | 209 | 0.296 |
| Annals of Thoracic Surgery | 208 | 0.295 |
| Journal of Infection | 199 | 0.282 |
| Journal of Hospital Infection | 198 | 0.281 |
| Anesthesiology | 198 | 0.281 |
| Pediatric Research | 193 | 0.274 |
| Current Opinion In Critical Care | 187 | 0.265 |
| Thrombosis And Haemostasis | 183 | 0.259 |
| American Journal of Emergency Medicine | 183 | 0.259 |
| British Journal of Anaesthesia | 180 | 0.255 |
| Journal of Applied Physiology | 179 | 0.254 |
| Anaesthesia And Intensive Care | 177 | 0.251 |
| American Journal of Obstetrics And Gynecology | 176 | 0.249 |
| Anaesthesist | 175 | 0.248 |
| World Journal of Gastroenterology | 174 | 0.247 |
| Hepato Gastroenterology | 173 | 0.245 |
| Bmc Infectious Diseases | 173 | 0.245 |
| Critical Care Clinics | 171 | 0.242 |
| Clinical Microbiology And Infection | 169 | 0.239 |
| Archives of Disease In Childhood | 169 | 0.239 |
| Infection | 167 | 0.237 |
| Annales Francaises D Anesthesie Et De Reanimation | 166 | 0.235 |
| Nephrology Dialysis Transplantation | 162 | 0.23 |
| American Journal of Physiology Lung Cellular And Molecular Physiology | 162 | 0.23 |
| Proceedings of The National Academy Of Sciences Of The United States Of America | 159 | 0.225 |
| American Journal of Physiology Heart And Circulatory Physiology | 159 | 0.225 |
| Nutrition | 157 | 0.222 |
| Journal of Endotoxin Research | 157 | 0.222 |
| European Journal of Pediatrics | 156 | 0.221 |
| American Journal of Perinatology | 155 | 0.22 |
| >=5 per year (75 journals ) | 26920 | 38.150 |
| International Journal of Antimicrobial Agents | 153 | 0.217 |
| Clinical Nutrition | 151 | 0.214 |
| Obstetrics And Gynecology | 150 | 0.213 |
| Minerva Anestesiologica | 149 | 0.211 |
| Thrombosis Research | 148 | 0.21 |
| European Journal of Pharmacology | 147 | 0.208 |
| Journal of Clinical Investigation | 145 | 0.205 |
| Cochrane Database of Systematic Reviews | 141 | 0.2 |
| Transfusion | 139 | 0.197 |
| International Immunopharmacology | 139 | 0.197 |
| Journal of Perinatology | 138 | 0.196 |
| Biochemical And Biophysical Research Communications | 138 | 0.196 |
| Bone Marrow Transplantation | 137 | 0.194 |
| Transplantation | 136 | 0.193 |
| European Journal of Cardio Thoracic Surgery | 136 | 0.193 |
| Clinical Orthopaedics And Related Research | 136 | 0.193 |
| British Journal of Pharmacology | 136 | 0.193 |
| Pediatric Surgery International | 135 | 0.191 |
| Clinical And Experimental Immunology | 133 | 0.188 |
| American Journal of Physiology Regulatory Integrative And Comparative Physiology | 133 | 0.188 |
| Kidney International | 132 | 0.187 |
| Inflammation Research | 132 | 0.187 |
| Inflammation | 130 | 0.184 |
| Blood Purification | 130 | 0.184 |
| Journal of Medical Microbiology | 129 | 0.183 |
| Journal of The American College Of Surgeons | 128 | 0.181 |
| Resuscitation | 126 | 0.179 |
| Medecine Et Maladies Infectieuses | 125 | 0.177 |
| Hepatology | 125 | 0.177 |
| Mediators Of Inflammation | 124 | 0.176 |
| Archives De Pediatrie | 124 | 0.176 |
| Journal of Vascular Surgery | 121 | 0.171 |
| Infection Control And Hospital Epidemiology | 121 | 0.171 |
| Journal of Urology | 120 | 0.17 |
| Journal of Maternal Fetal Neonatal Medicine | 120 | 0.17 |
| American Journal Of Physiology Gastrointestinal And Liver Physiology | 117 | 0.166 |
| Journal of Veterinary Internal Medicine | 115 | 0.163 |
| European Journal of Surgery | 114 | 0.162 |
| Pediatric Emergency Care | 113 | 0.16 |
| Journal of Thoracic And Cardiovascular Surgery | 113 | 0.16 |
| Chinese Medical Journal | 112 | 0.159 |
| Clinical Science | 111 | 0.157 |
| Cancer | 111 | 0.157 |
| American Journal of Physiology | 111 | 0.157 |
| Presse Medicale | 108 | 0.153 |
| Journal of Thrombosis And Haemostasis | 108 | 0.153 |
| Journal of Trauma And Acute Care Surgery | 106 | 0.15 |
| Journal of Clinical Oncology | 105 | 0.149 |
| Chirurg | 105 | 0.149 |
| Faseb Journal | 104 | 0.147 |
| Annals of Pharmacotherapy | 104 | 0.147 |
| Renal Failure | 103 | 0.146 |
| Internal Medicine | 103 | 0.146 |
| European Respiratory Journal | 103 | 0.146 |
| Current Opinion In Infectious Diseases | 102 | 0.145 |
| Wiener Klinische Wochenschrift | 101 | 0.143 |
| Life Sciences | 100 | 0.142 |
| Diagnostic Microbiology And Infectious Disease | 100 | 0.142 |
| Academic Emergency Medicine | 100 | 0.142 |
| Pharmacotherapy | 99 | 0.14 |
| Medicina Clinica | 99 | 0.14 |
| International Journal of Artificial Organs | 99 | 0.14 |
| American Journal of Physiology Endocrinology And Metabolism | 99 | 0.14 |
| American Journal of Kidney Diseases | 99 | 0.14 |
| Zentralblatt Fur Chirurgie | 98 | 0.139 |
| Revista Medica De Chile | 98 | 0.139 |
| Langenbecks Archives of Surgery | 98 | 0.139 |
| Injury International Journal of The Care Of The Injured | 98 | 0.139 |
| European Surgical Research | 98 | 0.139 |
| Contributions To Nephrology | 97 | 0.137 |
| Journal of Cardiothoracic And Vascular Anesthesia | 95 | 0.135 |
| Southern Medical Journal | 94 | 0.133 |
| Journal of Hepatology | 94 | 0.133 |
| Circulation | 94 | 0.133 |
| Journal of Pharmacology And Experimental Therapeutics | 93 | 0.132 |
| British Journal of Haematology | 93 | 0.132 |
| Anasthesiologie Intensivmedizin | 93 | 0.132 |
| Journal of Veterinary Emergency And Critical Care | 92 | 0.13 |
| Journal of Emergency Medicine | 92 | 0.13 |
| Deutsche Medizinische Wochenschrift | 92 | 0.13 |
| Journal of Gastrointestinal Surgery | 91 | 0.129 |
| European Journal Of Immunology | 91 | 0.129 |
| Medicina Intensiva | 90 | 0.128 |
| Journal of Paediatrics And Child Health | 89 | 0.126 |
| Journal of Chemotherapy | 89 | 0.126 |
| Anaesthesia | 89 | 0.126 |
| American Journal of Pathology | 89 | 0.126 |
| Surgical Infections | 88 | 0.125 |
| Journal of Tropical Pediatrics | 88 | 0.125 |
| Clinical Chemistry And Laboratory Medicine | 88 | 0.125 |
| American Journal of Respiratory Cell And Molecular Biology | 88 | 0.125 |
| American Journal of Medicine | 88 | 0.125 |
| Early Human Development | 87 | 0.123 |
| Pediatric Nephrology | 86 | 0.122 |
| Journal of Pediatric Hematology Oncology | 86 | 0.122 |
| Journal of Experimental Medicine | 86 | 0.122 |
| Journal of The Formosan Medical Association | 85 | 0.12 |
| Archives of Internal Medicine | 85 | 0.12 |
| American Journal of The Medical Sciences | 85 | 0.12 |
| Surgical Endoscopy and Other Interventional Techniques | 84 | 0.119 |
| Pediatrics International | 84 | 0.119 |
| Leukemia Lymphoma | 84 | 0.119 |
| Blood Coagulation Fibrinolysis | 84 | 0.119 |
| Saudi Medical Journal | 83 | 0.118 |
| Molecular Medicine | 83 | 0.118 |
| International Journal of Infectious Diseases | 83 | 0.118 |
| European Journal of Anaesthesiology | 83 | 0.118 |
| Journal of Burn Care Research | 82 | 0.116 |
| Free Radical Biology And Medicine | 82 | 0.116 |
| Journal of Clinical Endocrinology Metabolism | 80 | 0.113 |
| Digestive Diseases And Sciences | 79 | 0.112 |
| Pancreas | 77 | 0.109 |
| Internist | 77 | 0.109 |
| Annals of The New York Academy of Sciences | 77 | 0.109 |
| American Journal of Infection Control | 77 | 0.109 |
| Journal of Heart And Lung Transplantation | 76 | 0.108 |
| Journal of Arthroplasty | 76 | 0.108 |
| Indian Journal of Pediatrics | 75 | 0.106 |
| Surgery Today | 74 | 0.105 |
| Liver Transplantation | 74 | 0.105 |
| Annals of Oncology | 74 | 0.105 |
| Gut | 73 | 0.103 |
| Anasthesiologie Intensivmedizin Notfallmedizin Schmerztherapie | 73 | 0.103 |
| Colorectal Disease | 72 | 0.102 |
| Biology of The Neonate | 72 | 0.102 |
| Asaio Journal | 72 | 0.102 |
| International Journal of Colorectal Disease | 71 | 0.101 |
| Annals of The Royal College of Surgeons Of England | 71 | 0.101 |
| Advances In Experimental Medicine And Biology | 71 | 0.101 |
| Vaccine | 70 | 0.099 |
| Medical Hypotheses | 69 | 0.098 |
| Seminars In Respiratory And Critical Care Medicine | 67 | 0.095 |
| Current Opinion In Clinical Nutrition And Metabolic Care | 67 | 0.095 |
| Clinical Transplantation | 67 | 0.095 |
| Archives Of Disease In Childhood Fetal And Neonatal Edition | 67 | 0.095 |
| Annals Of Emergency Medicine | 67 | 0.095 |
| Turkish Journal Of Pediatrics | 66 | 0.094 |
| Journal Of The American Society Of Nephrology | 66 | 0.094 |
| American Journal Of Veterinary Research | 66 | 0.094 |
| Neonatology | 65 | 0.092 |
| Journal Of Pediatric Gastroenterology And Nutrition | 65 | 0.092 |
| Indian Pediatrics | 65 | 0.092 |
| Cardiovascular Research | 65 | 0.092 |
| Annals Of Hematology | 65 | 0.092 |
| Surgical Clinics Of North America | 64 | 0.091 |
| Immunobiology | 64 | 0.091 |
| Clinics In Chest Medicine | 64 | 0.091 |
| Urology | 63 | 0.089 |
| British Medical Journal | 63 | 0.089 |
| American Journal Of Hematology | 63 | 0.089 |
| Acta Chirurgica Belgica | 63 | 0.089 |
| Fems Immunology And Medical Microbiology | 62 | 0.088 |
| Equine Veterinary Journal | 62 | 0.088 |
| Acta Clinica Belgica | 62 | 0.088 |
| >=2 per year (229 journals) | 42072 | 59.622 |

B. Global annual number of sepsis articles published in the most five popular journals (Critical Care Medicine, Shock, Intensive Care Medicine Critical Care and PLos One).

| Year | Critical Care Medicine | Shock | Intensive Care Medicine | Critical Care | PloS One |
| --- | --- | --- | --- | --- | --- |
| 1984 | 6 | 0 | 1 | 0 | 0 |
| 1985 | 13 | 0 | 0 | 0 | 0 |
| 1986 | 7 | 0 | 1 | 0 | 0 |
| 1987 | 11 | 0 | 3 | 0 | 0 |
| 1988 | 11 | 0 | 8 | 0 | 0 |
| 1989 | 23 | 0 | 2 | 0 | 0 |
| 1990 | 40 | 0 | 11 | 0 | 0 |
| 1991 | 74 | 0 | 18 | 0 | 0 |
| 1992 | 58 | 0 | 20 | 0 | 0 |
| 1993 | 80 | 0 | 13 | 0 | 0 |
| 1994 | 69 | 77 | 27 | 0 | 0 |
| 1995 | 86 | 62 | 24 | 0 | 0 |
| 1996 | 104 | 74 | 49 | 0 | 0 |
| 1997 | 79 | 55 | 39 | 0 | 0 |
| 1998 | 80 | 62 | 48 | 0 | 0 |
| 1999 | 124 | 68 | 52 | 8 | 0 |
| 2000 | 196 | 79 | 99 | 11 | 0 |
| 2001 | 126 | 78 | 66 | 6 | 0 |
| 2002 | 158 | 81 | 64 | 27 | 0 |
| 2003 | 162 | 90 | 88 | 22 | 0 |
| 2004 | 149 | 92 | 82 | 41 | 0 |
| 2005 | 163 | 88 | 71 | 66 | 0 |
| 2006 | 150 | 79 | 79 | 96 | 0 |
| 2007 | 163 | 91 | 90 | 71 | 5 |
| 2008 | 178 | 103 | 97 | 85 | 9 |
| 2009 | 164 | 94 | 77 | 131 | 20 |
| 2010 | 127 | 101 | 86 | 143 | 41 |
| 2011 | 107 | 96 | 63 | 140 | 69 |
| 2012 | 121 | 93 | 62 | 124 | 153 |
| 2013 | 150 | 79 | 53 | 162 | 221 |
| 2014 | 124 | 79 | 47 | 182 | 200 |
| Total | 3103 | 1721 | 1440 | 1315 | 718 |
| Average Annual Growth Rate (%) | 10.622 | 0.128 | 13.694 | 23.159 | 69.381 |
| R- Square | 0.6986 | 0.4649 | 0.6776 | 0.9397 | 0.8842 |
| P-Value | < 0.0001 | 0.0007 | < 0.0001 | < 0.0001 | 0.0005 |

C. The China’s annually sepsis publications in the five globally most popular journals (Critical Care Medicine, Shock, Intensive Care Medicine Critical Care and PLos One).

| year | Critical Care Medicine | Shock | Intensive Care Medicine | Critical Care | PloS One |
| --- | --- | --- | --- | --- | --- |
| 1995 | 0 | 1 | 0 | 0 | 0 |
| 1996 | 0 | 0 | 0 | 0 | 0 |
| 1997 | 0 | 0 | 0 | 0 | 0 |
| 1998 | 0 | 0 | 0 | 0 | 0 |
| 1999 | 0 | 0 | 0 | 0 | 0 |
| 2000 | 0 | 1 | 1 | 0 | 0 |
| 2001 | 2 | 4 | 0 | 0 | 0 |
| 2002 | 2 | 4 | 0 | 0 | 0 |
| 2003 | 0 | 3 | 1 | 0 | 0 |
| 2004 | 0 | 3 | 1 | 0 | 0 |
| 2005 | 0 | 1 | 0 | 0 | 0 |
| 2006 | 1 | 1 | 0 | 0 | 0 |
| 2007 | 2 | 5 | 0 | 2 | 1 |
| 2008 | 4 | 5 | 1 | 2 | 0 |
| 2009 | 2 | 9 | 2 | 3 | 0 |
| 2010 | 3 | 7 | 1 | 3 | 3 |
| 2011 | 3 | 8 | 1 | 9 | 6 |
| 2012 | 3 | 8 | 1 | 6 | 23 |
| 2013 | 2 | 8 | 1 | 13 | 29 |
| 2014 | 4 | 12 | 1 | 20 | 37 |
| Total | 28 | 80 | 11 | 58 | 99 |
| Average Annual Growth Rate (%) | 5.477 | 13.972 | 0.000 | 38.950 | 67.505 |
| R- Square | 0.4046 | 0.7839 | 0.1853 | 0.7844 | 0.8394 |
| P-Value | 0.0145 | < 0.0001 | 0.1092 | 0.0034 | 0.0014 |

Supplementary Appendix 6. China’s high impact institutions, growth trend of NSFC.

A. Number of articles, citations and h-index from top 20 highest impact institutions in China.

| Organization | Publications | Percentage (n=2542) | Citations | H-index | Province |
| --- | --- | --- | --- | --- | --- |
| Zhejiang University | 175 | 6.884 | 1880 | 19 | Zhejiang |
| Nanjing University | 148 | 5.822 | 1644 | 24 | Nanjing |
| Third Military Medical University | 142 | 5.586 | 1381 | 20 | Chongqing |
| Fudan University | 124 | 4.878 | 1585 | 19 | Shanghai |
| Second Military Medical University | 115 | 4.524 | 1350 | 19 | Shanghai |
| Shanghai Jiao Tong University | 114 | 4.485 | 1277 | 17 | Shanghai |
| Chinese People S Liberation Army General Hospital | 114 | 4.485 | 1240 | 21 | Peking |
| University of Hong Kong | 108 | 4.249 | 2255 | 26 | Hong Kong |
| Capital Medical University | 105 | 4.130 | 996 | 15 | Peking |
| Chinese University of Hong Kong | 103 | 4.052 | 3123 | 29 | Hong Kong |
| Prince of Wales Hospital | 86 | 3.383 | 2734 | 27 | Hong Kong |
| Chinese Academy of Medical Sciences Peking Union Medical College | 86 | 3.383 | 1379 | 17 | Peking |
| Peking University | 83 | 3.265 | 1639 | 18 | Peking |
| Central South University | 81 | 3.186 | 964 | 17 | Hunan |
| Huazhong University of Science Technology | 68 | 2.675 | 759 | 17 | Hubei |
| Sun Yat Sen University | 67 | 2.635 | 643 | 12 | Guangdong |
| Sichuan University | 67 | 2.635 | 466 | 12 | Szechwan |
| Fourth Military Medical University | 63 | 2.478 | 978 | 17 | Shaanxi |
| Chinese Academy of Sciences | 61 | 2.400 | 1198 | 18 | Peking |
| China Medical University | 59 | 2.321 | 478 | 12 | Liaoning |
| Total | 1969 | 77.459 | 27969 | / | / |

NSFC: National Natural Science Foundation of China.

B. Annual amount of money and projects from NSFC between 1984 and 2014.

| Year | NSFC Amount (×10,000 RMB) | Projects |
| --- | --- | --- |
| 1986 | 2 | 1 |
| 1987 | 3 | 1 |
| 1988 | 3 | 1 |
| 1989 | 0 | 0 |
| 1990 | 2 | 1 |
| 1991 | 0 | 0 |
| 1992 | 0 | 0 |
| 1993 | 0 | 0 |
| 1994 | 12 | 2 |
| 1995 | 9 | 1 |
| 1996 | 0 | 0 |
| 1997 | 0 | 0 |
| 1998 | 0 | 0 |
| 1999 | 13 | 1 |
| 2000 | 18 | 1 |
| 2001 | 97 | 2 |
| 2002 | 61 | 2 |
| 2003 | 58 | 3 |
| 2004 | 105 | 5 |
| 2005 | 298 | 12 |
| 2006 | 127 | 5 |
| 2007 | 103 | 4 |
| 2008 | 544 | 14 |
| 2009 | 431 | 16 |
| 2010 | 862 | 33 |
| 2011 | 3048 | 55 |
| 2012 | 2431 | 50 |
| 2013 | 2252 | 50 |
| 2014 | 2,956 | 56 |
| Total | 13435 | 316 |
| Average Annual Growth Rate (%) | 29.778 | 15.461 |
| R- Square | 0.4821 | 0.5687 |
| P-Value | < 0.0001 | < 0.0001 |

NSFC: National Natural Science Foundation of China.

Supplementary Appendix 7. Fifteen most popular journalsinthe USA, Germany, England, France, Japan and China.

| Rank | United States (IF) | N | Germany (IF) | N | England (IF) | N | France (IF) | N | Japan(IF) | N | China (IF) | N |
| --- | --- | --- | --- | --- | --- | --- | --- | --- | --- | --- | --- | --- |
| 1 | Crit Care Med (7.422) | 1383 | Crit Care Med (7.422) | 369 | Crit Care Med (7.422) | 229 | Crit Care Med (7.422) | 331 | Crit Care Med (7.422) | 113 | Chin Med J (Engl) (0.957) | 110 |
| 2 | Shock (3.048) | 848 | Intensive Care Med (10.125) | 280 | Intensive Care Med (10.125) | 167 | Intensive Care Med (10.125) | 300 | Shock (3.048) | 97 | PloS One (3.057) | 99 |
| 3 | J Trauma (2013:2.961) | 463 | Shock (3.048) | 219 | Br J Surg (5.596) | 107 | Crit Care (4.950) | 171 | Intern Med (0.832) | 89 | Shock (3.048) | 81 |
| 4 | J Surg Res (2.198) | 407 | Crit Care (4.950) | 207 | Crit Care (4.950) | 99 | Ann Fr Anesth (0.917) | 156 | J Surg Res (2.198) | 73 | World J Gastroentero (2.787) | 67 |
| 5 | J Immunol (4.985) | 378 | Anaesthesist (0.964) | 162 | Arch Dis Child (3.231) | 95 | Med Maladies Infect (1.422) | 106 | J Infect Chemother (1.425) | 50 | IntImmunopharmacol(2.551) | 66 |
| 6 | Chest (5.940） | 351 | Chirurg (0.630) | 96 | Lancet (44.002) | 93 | Arch Pediatrie (0.401) | 102 | Hepato-gastroenterology (0.792) | 50 | J Surg Res (2.198) | 62 |
| 7 | Pediatrics（5.196） | 311 | ZentralblChir (0.638) | 85 | J AntimicrobChemother (4.919) | 69 | Presse Med (1.015) | 97 | Surg Today (1.329) | 44 | Crit Care (4.950) | 58 |
| 8 | Arch Surg (2014:4.926) | 307 | Dtsch Med Wochenschr (0.503) | 82 | Br J Anaesth (5.616) | 63 | Am J RespirCrit Care Med (13.118) | 63 | J Immunol (4.985) | 39 | Inflammation (2.681) | 40 |
| 9 | Crit Care (4.950) | 294 | AnasthesiologieIntensivmedizin (1.09) | 82 | J Hosp Infect (2.655) | 61 | Shock (3.048) | 62 | Thromb Res (2.320) | 37 | Crit Care Med (7.422) | 36 |
| 10 | Surgery (3.309) | 266 | J Immun (4.985) | 76 | Shock (3.048) | 54 | Chest (5.940） | 55 | J Trauma (2013:2.961) | 36 | Burns (1.940) | 33 |
| 11 | Infect Immun (3.603) | 266 | Internist (0.336) | 69 | PloS One (3.057) | 53 | Anesthesiology (5.264) | 42 | Crit Care (4.950) | 36 | ExpTher Med (1.280) | 28 |
| 12 | PloS One (3.057) | 260 | Langenbecks Arch Surg (2.149) | 68 | J PediatrSurg (1.733) | 50 | Clin Infect Dis (8.736) | 41 | TherApher Dial (1.477) | 34 | Mol Med Rep (1.559) | 23 |
| 13 | Am J RespirCrit Care Med (13.118) | 249 | AnasthIntensivNotf (0.325) | 68 | Br J Pharmacol (5.259) | 50 | Revue Med Interne (0.905) | 40 | Int J Hematol (1.846) | 30 | MediatInflamm (3.418) | 23 |
| 14 | Pediatr Infect Dis J (2.587) | 240 | Infection (2.294) | 62 | Ann R CollSurgEngl (1.332) | 48 | PloS One (3.057) | 39 | Intensive Care Med (10.125) | 30 | J Trauma (2013:2.961) | 21 |
| 15 | Ann Surg (8.569) | 233 | PloS One (3.057) | 51 | Anaesthesia (3.794) | 45 | JAMA-J Am Med Assoc(37.684) | 35 | AnesthAnalg (3.827) | 30 | Inflamm Res (2.557) | 21 |
